# Iloprost, a Prostacyclin Analogue, Alleviates Oxidative Stress and Improves Development of Parthenogenetic Porcine Embryos via Nrf2/Keap1 Signaling

**DOI:** 10.3390/antiox14121493

**Published:** 2025-12-12

**Authors:** Eun Young Choi, Kyungjun Uh, Seol-Bin Lee, Pil-Soo Jeong, Hyo-Gu Kang, Se-Been Jeon, Ji Hyeon Yun, Hee-Chang Son, Kyung-Seob Lim, You Jeong An, Sun-Uk Kim, Seong-Keun Cho, Bong-Seok Song

**Affiliations:** 1 Futuristic Animal Resource & Research Center (FARRC), Korea Research Institute of Bioscience and Biotechnology (KRIBB), Cheongju 28116, Republic of Korea; comyou6@kribb.re.kr (E.Y.C.); kjuh@kribb.re.kr (K.U.); snowbean@kribb.re.kr (S.-B.L.); spectrum@kribb.re.kr (P.-S.J.); kogd1887@kribb.re.kr (H.-G.K.); dialdial220@kribb.re.kr (S.-B.J.); yoonzzih@kribb.re.kr (J.H.Y.); son1989@kribb.re.kr (H.-C.S.); dvmlim96@kribb.re.kr (K.-S.L.); sunuk@kribb.re.kr (S.-U.K.); 2 Department of Animal Science, College of Natural Resources & Life Science, Pusan National University, Miryang 50463, Republic of Korea; 3 Department of Advanced Bioconvergence, University of Science and Technology (UST), Daejeon 34113, Republic of Korea; 4 Department of Animal Science, College of Agriculture, Life & Environment Science, Chungbuk National University, Cheongju 28644, Republic of Korea; 5 Department of Animal Science and Biotechnology, College of Agriculture and Life Science, Chungnam National University, Daejeon 34134, Republic of Korea; 6 National Primate Research Centre, Korea Research Institute of Bioscience and Biotechnology (KRIBB), Cheongju 28116, Republic of Korea; ayj0610@kribb.re.kr; 7 Department of Functional Genomics, University of Science and Technology (UST), Daejeon 34113, Republic of Korea; 8 Department of Animal Science, College of Natural Resources and Life Science, Life and Industry Convergence Research Institute (LICRI), Pusan National University, Gyeongsangnam-do, Miryang 50463, Republic of Korea

**Keywords:** Iloprost, porcine embryo, oxidative stress, Nrf2/Keap1, antioxidant genes

## Abstract

Background: Prostacyclin (PGI_2_), an abundantly produced bioactive lipid by oviductal epithelial cells, supports preimplantation embryo development by buffering oxidative stress. However, the mechanism linking PGI_2_ signaling to embryonic redox control remains unclear. We investigated whether Iloprost (Ilo), a stable PGI_2_ analogue, enhances preimplantation embryo development by alleviating oxidative stress via activation of the Nrf2/Keap1 pathway, and whether these effects depend on Nrf2 activity using the inhibitor brusatol. Methods: Porcine embryos were treated with Ilo to model oviductal PGI_2_ signaling during in vitro culture. Developmental competence was evaluated by cleavage and blastocyst formation rates, and blastocyst quality by total cell number and TUNEL assays. Oxidative status was quantified by fluorescence detection of reactive oxygen species (ROS), and Nrf2 activation was assessed by nuclear localization and antioxidant-related gene expression. Results: Embryos treated with Ilo showed significantly increased blastocyst formation, reduced ROS, and upregulated antioxidant genes. Immunofluorescence confirmed increased nuclear translocation of Nrf2, indicating activation of the Nrf2/Keap1 signaling pathway. In contrast, embryos treated with brusatol showed reduced blastocyst formation, increased ROS, and downregulated antioxidant-related gene expression, whereas co-treatment with Ilo reversed these effects. Conclusions: This study demonstrates that PGI_2_ protects embryos by activating Nrf2/Keap1 signaling, establishing this axis as a key antioxidant defense during embryonic development and highlighting its potential to improve embryo culture systems.

## 1. Introduction

The oviduct plays a crucial role in early embryonic development by providing a suitable environment for fertilization and preimplantation development [1]. A complex regulatory mechanism in the oviductal tract is essential for successful embryonic development and implantation in the uterus [2]. The oviductal tract provides an appropriate microenvironment for the embryo as it travels to the uterus, and three key elements must be properly regulated: secretory cells that produce oviductal fluid, the beating movement of ciliated cells that induce the flow of oviductal fluid, and the contractile activity of smooth muscle cells that assist this flow [3]. As the embryo moves toward the uterus, it interacts with various molecules secreted by oviductal cells, but the specific molecules and their biological functions are not fully defined [4,5,6].

Among these secreted molecules, prostacyclin (PGI_2_), a bioactive lipid of the prostanoid family, has gained particular attention. PGI_2_ is synthesized via the arachidonic acid metabolic pathway and is produced mainly by endothelial and oviductal epithelial cells, especially by secretory cells [7,8,9]. In the human fallopian tube, PGI_2_ facilitates embryo transport and supports developmental competence through IP receptor signaling, which activates the production of cyclic AMP (cAMP) and downstream signaling pathways that regulate anti-inflammatory responses, platelet aggregation, smooth muscle relaxation, and cell proliferation [10,11].

Studies using animal embryo models have shown that PGI_2_ and its stable analogue, Iloprost (Ilo), enhance implantation rates, improve oocyte maturation, and increase blastocyst formation [12,13,14,15]. Notably, these effects are closely associated with the mitigation of oxidative stress during early embryonic development, but the underlying mechanisms remain unclear.

Reactive oxygen species (ROS), including superoxide anions, hydrogen peroxide, hydroxyl radicals, and others, are primarily produced as byproducts of mitochondrial respiration [16]. These substances are recognized as significant endogenous stress factors that have the capacity to disrupt cellular homeostasis and compromise embryonic development [17]. Notably, ROS exhibits a dual role in early embryonic development. At physiologically low levels, ROS act as essential signaling molecules involved in cell-to-cell communication and differentiation [18]. However, when produced in excess, they have been shown to induce oxidative stress and cause cellular damage by affecting lipids, proteins, and DNA, thereby reducing embryonic viability and developmental potential [19]. In vitro culture (IVC) condition often exacerbates ROS accumulation due to elevated oxygen concentration, light exposure, and medium components, leading to impaired blastocyst formation and quality [20,21,22]. Conversely, the in vivo oviductal milieu maintains low ROS levels by secreting antioxidant enzymes and protective factors, providing a favorable environment for embryo development [23]. However, the molecular mechanisms underlying this antioxidant protection remain poorly understood.

The nuclear factor erythroid-2-related factor 2 (Nrf2)/Kelch-like ECH-associated protein 1 (Keap1) signaling pathway is a key regulator of antioxidant defense [24]. Under normal physiological conditions, Keap1 binds to Nrf2 in the cytoplasm, promoting its ubiquitination and subsequent degradation by the proteasome. However, oxidative stress modifies Keap1 and allows Nrf2 to translocate to the nucleus [25]. Within the nucleus, Nrf2 binds to antioxidant response elements (AREs) located in the promoter regions of target genes, inducing the transcription of key antioxidant-related genes such as glutathione peroxidase (*GPX*), superoxide dismutase (*SOD*), catalase (*CAT*), and heme oxygenase-1 (*HO-1*) [26]. Although PGI_2_ has been reported to improve embryo development and implantation, whether its effects are mediated through Nrf2/keap1 activation is unknown [27].

Therefore, the aim of this study was to investigate whether Ilo, a stable analogue of PGI_2_, enhances developmental competence in porcine embryos by alleviating oxidative stress through activation of the Nrf2/Keap1 signaling pathway. By using porcine embryos as a large-animal model that shares key physiological features with humans, this work provides mechanistic insight into how PGI_2_ signaling supports early embryonic development.

## 2. Materials and Methods

### 2.1. Chemicals

Unless otherwise specified, all chemicals and reagents used in this study were purchased from Sigma-Aldrich (St. Louis, MO, USA).

### 2.2. Oocyte Collection and In Vitro Maturation (IVM)

Porcine ovaries obtained from a local slaughterhouse were transported to the laboratory in 0.9% saline containing 0.75 μg/mL benzyl-penicillin potassium (Wako, Osaka, Japan) and 0.5 μg/mL streptomycin sulfate, while maintaining the solution at 38.5 °C during transport. Cumulus–oocyte complexes (COCs) were obtained by aspirating follicular fluid from 3–8 mm follicles with an 18-gauge needle. COCs exhibiting a homogeneous ooplasm and well-preserved cumulus cell layers were chosen, then rinsed in saline containing 1 mg/mL BSA. IVM was performed in two sequential stages: phase I (0–22 h) in Medium-199 enriched with porcine follicular fluid and maturation supplements, followed by phase II (22–44 h) in the same base medium without hormonal additives. During the maturation phase, COCs were cultured in an atmosphere of 5% CO_2_ at 38.5 °C using a Heracell CO_2_ incubator (Thermo Fisher Scientific, Waltham, MA, USA). Following maturation, cumulus cells were removed by treating the COCs with 0.1% hyaluronidase. Oocytes that had reached the Metaphase II stage were recognized by the appearance of an extruded polar body when examined under an Olympus SZ61 stereomicroscope (Olympus Corporation, Tokyo, Japan).

### 2.3. Parthenogenetic Activation (PA) and IVC

Matured oocytes were activated parthenogenetically by incubating them for 5 min in ionomycin (15 μM) prepared in a DPBS solution (Gibco, Carlsbad, CA, USA) that also contained 60 μg/mL gentamicin, 75 μg/mL streptomycin, and 4 mg/mL BSA, and the procedure was conducted under light-protected conditions. After being rinsed several times, the activated oocytes were placed in PZM-3 supplemented with 5 μg/mL cytochalasin B and 2 mM 6-DMAP and kept in this medium for approximately 4 h. The embryos were subsequently placed into freshly prepared PZM-3 medium containing BSA at a concentration of 4 mg/mL and maintained for 144 h inside a Heracell CO_2_ incubator (Thermo Fisher Scientific, Waltham, MA, USA) set to 38.5 °C with a 5% CO_2_ atmosphere. Developmental progression was evaluated at 48 h (cleavage) and 144 h (blastocyst formation) post-activation.

### 2.4. Chemical Treatment

Iloprost (Cayman Chemical, cat# 18215, Ann Arbor, MI, USA) stock solutions were prepared in DMSO and incorporated into IVC medium at a working concentration of 1 μM, determined from prior optimization [28]. Nrf2 inhibition was achieved using brusatol (Sigma-Aldrich, cat# SML1868), also solubilized in DMSO and serially diluted into IVC medium at 0, 10, 50, or 100 nM. Dose–response screening identified 50 nM brusatol as optimal for inhibiting Nrf2 signaling without cytotoxicity in subsequent mechanistic studies.

### 2.5. Apoptosis Detection Through TdT-Mediated dUTP Labeling (TUNEL) Assay

Apoptotic nuclei were visualized using a commercial TUNEL-based detection kit (Roche, Basel, Switzerland). For sample preparation, blastocysts were fixed in 4% paraformaldehyde at 4 °C overnight and subsequently washed three times with DPBS supplemented with 0.1% PVA (PVA-PBS). Cell membranes were permeabilized in 1% Triton X-100 (*v*/*v*) for 1 h at room temperature. After permeabilization, embryos were rinsed three times with PVA-PBS and subsequently incubated with fluorescein-dUTP together with TdT at 38.5 °C for 1 h in the dark. Following labeling, blastocysts were washed again in PVA-PBS, stained with a DAPI solution (1.5 μg/mL; Vector Laboratories, Newark, CA, USA), and then mounted for imaging on a Leica DMi8 microscope(Leica Microsystems, Wetzlar, Germany) to quantify apoptotic nuclei.

### 2.6. Measurement of Intracellular ROS and Glutathione (GSH) Levels

To assess cellular redox status, measurements of CM-H_2_DCFDA and CMF_2_HC fluorescence (Invitrogen, Carlsbad, CA, USA) were performed, providing estimates of oxidative and reduced states. Embryos at the 4-cell stage (Day 2) and blastocysts (Day 6) were washed three times in PVA-PBS prior to staining. For ROS measurement, samples were incubated in PVA-PBS containing 5 μM CM-H_2_DCFDA for 20 min at room temperature, whereas GSH was detected by exposing embryos to 10 μM CMF_2_HC for 10 min under the same conditions. After dye loading, embryos were thoroughly rinsed with PVA-PBS to eliminate residual fluorescent probe. Fluorescent images were obtained on a Leica DMi4000B platform (Leica Microsystems, Wetzlar, Germany), which was fitted with UV filters for ROS (460 nm) and GSH (370 nm) detection. Fluorescence intensity was quantified with ImageJ software (version 1.47; NIH), and values were normalized to the mean intensity of control embryos.

### 2.7. Immunocytochemistry

Blastocysts were fixed by placing them in 4% paraformaldehyde at 4 °C overnight, after which they were washed three times in DPBS supplemented with 0.1% PVA (PVA-PBS). Membrane permeabilization was achieved by incubating samples in 1% Triton X-100 (*v*/*v*) for 1 h at room temperature. Following permeabilization, embryos were transferred to a blocking solution consisting of DPBS containing 2 mg/mL BSA and 0.05% Tween-20 for 1 h. Primary antibodies against Nrf2 (1:200; ab31163, Abcam, Cambridge, UK) and Keap1 (1:200; ab226997, Abcam) were then applied, and samples were kept at 4 °C overnight. The next day, blastocysts were washed three times with DPBS containing 0.05% Tween-20 and re-incubated in blocking buffer for 1 h. Subsequently, Alexa Fluor 488–conjugated goat anti-rabbit IgG (1:200; Thermo Fisher Scientific) was added for 1 h at room temperature. After final washes in DPBS with 0.05% Tween-20, embryos were mounted on glass slides with DAPI for nuclear counterstaining. Fluorescence images were acquired using a Leica DMi8 microscope, and signal intensity was quantified with ImageJ software, normalized to the mean area of control blastocysts.

### 2.8. Real-Time Quantitative PCR (qPCR) Analysis

Polyadenylated mRNA was captured from blastocysts with the Dynabeads mRNA Direct Kit (Invitrogen, Carlsbad, CA, USA), following the procedures provided by the supplier. Embryos were initially disrupted in lysis/binding buffer at room temperature for approximately 5 min. Subsequently, Dynabeads oligo(dT)25 (30 µL) were introduced to selectively bind polyadenylated RNA. mRNA-bound Dynabeads were retrieved on a magnetic stand and then rinsed sequentially with Washing Buffer A, followed by Washing Buffer B supplied in the kit to eliminate residual impurities. Purified mRNA was released from the beads in 7 μL of Tris buffer and directly used for cDNA synthesis. cDNA synthesis was carried out with the PrimeScript RT Reagent Kit containing gDNA Eraser (Takara Bio Inc., Shiga, Japan), following the protocol provided by the supplier. The synthesized cDNA was subsequently utilized for qPCR analysis. Amplification reactions were carried out on an Mx3000P real-time system (Agilent, Santa Clara, CA, USA) with SYBR Premix Ex Taq (Takara Bio Inc.) as the reaction mix. qPCR amplification was conducted under the following program: samples were first heated to 95 °C for 5 min, after which they underwent 40 repetitions of a two-step cycle consisting of 95 °C for 20 s and 60 °C for 20 s. The nucleotide sequences of all primers employed in this work are provided in Table S1.

### 2.9. Experimental Design

In experiment 1, the impact of Ilo on the in vitro development of porcine PA embryos was assessed. A total of 231 PA embryos were cultured in IVC medium containing either 0 or 1 μM Ilo for the entire 6-day IVC period, with the control group receiving an equivalent volume of vehicle (0.1% DMSO). These embryos were utilized across at least three independent replicates. Post-culture, 93 Day-6 blastocysts were evaluated in triplicate for the number and percentage of TUNEL-positive cells. Additionally, mRNA expression levels of genes related to developmental potential and apoptosis were analyzed in Day-6 blastocysts from five separate replicates. Experiment 2 aimed to investigate the antioxidative properties of Ilo. Intracellular levels of ROS and GSH were quantified in 99 Day-2 embryos and 93 Day-6 blastocysts. These measurements were performed on samples treated with or without 1 μM Ilo, involving three independent replicates. For experiment 3, the influence of Ilo on the Nrf2/Keap1 pathway was examined. Immunofluorescence analysis was conducted on Day-6 blastocysts (67 for Nrf2 and 70 for Keap1, each from three independent replicates) to determine the abundance of Nrf2 and Keap1 following treatment with or without 1 μM Ilo. Furthermore, the mRNA expression levels of Nrf2/Keap1 pathway downstream target genes were analyzed from three independent replicates. Experiment 4 focused on the effects of brusatol (a selective Nrf2 inhibitor) on PA embryo development. A total of 138 embryos were cultured in IVC medium containing either 0 or 50 nM brusatol throughout the IVC period. Three independent replicates were performed. Subsequently, 106 Day-6 blastocysts were used, in triplicate, to quantify the number and percentage of TUNEL-positive cells. Experiment 5 explored the combined effects of brusatol and Ilo co-treatment. A large cohort of 681 embryos was cultured across seven independent replicates, exposed to various treatment groups for the full IVC period. Following culture, 148 Day-6 blastocysts were processed, in triplicate, for the assessment of TUNEL-positive cell numbers and ratios. Additionally, the mRNA expression levels of apoptosis-related genes were determined from six distinct replicates. In experiment 6, the antioxidative impact of brusatol and its modulation by Ilo co-treatment were evaluated. Intracellular ROS and GSH levels were measured in 139 Day-2 embryos and 83 Day-6 blastocysts, with all analyses conducted in three independent replicates. Lastly, experiment 7 investigated the effects of brusatol and Ilo co-treatment on the Nrf2/Keap1 pathway. Immunofluorescence analysis for Nrf2 and Keap1 abundance was performed on 155 Day-6 blastocysts (for Nrf2) from three independent replicates. The mRNA expression levels of relevant downstream target genes were also analyzed, drawing from three independent replicates.

### 2.10. Statistical Analysis

Each experiment was independently carried out at least three times. In the figure legends, R represents the number of repeated experiments, and n indicates the number of biological samples. Data are shown as the mean ± standard error of the mean (SEM). For comparisons among three or more groups, one-way analysis of variance was conducted, followed by Duncan’s multiple range test. Differences between the two groups were evaluated using Student’s *t*-test. Statistical significance was defined as *p* < 0.05.

## 3. Results

### 3.1. Effects of Ilo on the Developmental Competence of Porcine PA Embryos

**Figure 1:**
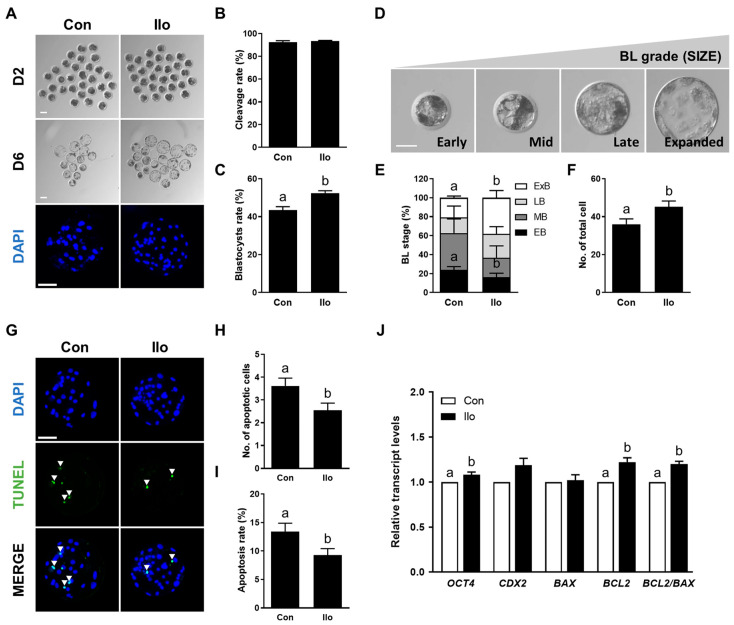
Effects of Iloprost (Ilo) treatment on early development of parthenogenetically activated porcine embryos. (**A**) Representative bright-field images (top and middle, scale bar = 100 μm) and nuclear-stained images (bottom, scale bar = 100 μm) of embryos at day 2 (cleaved embryos) and day 6 (blastocysts) after parthenogenetic activation (PA) with or without 1 μm Ilo. (**B**) Cleavage rate and (**C**) blastocyst formation rates of embryos from control and Ilo-treated groups after PA (R = 3; Con; n = 112, Ilo; n = 119). (**D**) Classification of blastocysts based on morphological stage: early blastocyst (EB), middle blastocyst (MB), late blastocyst (LB), and expanded blastocyst (ExB). Bar = 50 μm. (**E**) Proportions of different stages of blastocysts (R = 4; Con; n = 64, Ilo; n = 81). (**F**) Quantification of total cell numbers in the control and Ilo-treated groups (R = 3; Con; n = 112, Ilo; n = 119). (**G**) Representative images of terminal deoxynucleotidyl transferase-mediated dUTP-digoxygenin staining of blastocysts on day 6. Embryos were subjected to TUNEL (green, white arrow) and nuclear staining (blue). Bar = 100 μm. (**H**,**I**) Quantification of the number and proportion of apoptotic cells in the indicated groups (R = 3; Con; n = 47, Ilo; n = 46). (**J**) Relative expression levels of developmental potential and apoptosis-related genes and *BCL2*/*BAX* ratio in day 6 blastocysts with and without Ilo treatment (R = 5). Significant differences are represented by different letters (*p* < 0.05).

To evaluate the effects of Ilo on porcine embryonic development, PA embryos were cultured in an IVC medium treated with 1 μM Ilo for 6 days (Figure 1A). No significant difference was observed in cleavage rate between the embryos treated with Ilo and the control group (Figure 1A,B, Table S2). However, the blastocyst formation rate was significantly increased in embryos treated with Ilo (Figure 1A,C, Table S2). Morphological assessment of blastocysts revealed a significantly higher proportion of expanded blastocysts in the Ilo-treated group compared with controls (Figure 1D,E, Table S3). Consistent with these findings, the total cell number of blastocysts was significantly increased by Ilo treatment (Figure 1A,F, Table S2). TUNEL assay revealed that both the number and rate of apoptotic cells were lower in the Ilo-treated group compared with controls (Figure 1G–I, Table S4). Gene expression analysis further supported the improved developmental potential of embryos treated with Ilo, showing increased transcript levels of *OCT4* (Figure 1J). With respect to apoptosis-related transcripts, the level of *BAX* remained unchanged following Ilo exposure, whereas a pronounced increase in *BCL2* expression elevated the *BCL2*/*BAX* ratio, indicating enhanced anti-apoptotic activity (Figure 1J). Together, these results suggest that Ilo supplementation enhances blastocyst quality by promoting cell proliferation and reducing apoptosis.

### 3.2. Ilo Regulates Intracellular ROS and GSH Levels in Porcine PA Embryos

**Figure 2:**
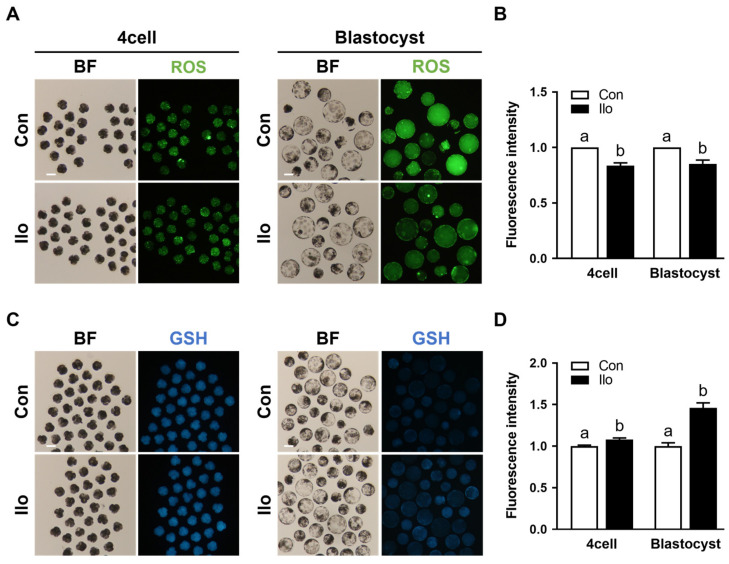
Effects of Iloprost (Ilo) treatment on intracellular ROS and GSH levels in porcine PA embryos. (**A**) Representative fluorescence images of day 2 embryos and day 6 blastocysts stained with CM-H2DCFDA for ROS detection in the control and Ilo-treated group. Scale bar = 100 μm. (**B**) Quantification of ROS fluorescence intensity in day 2 embryos and day 6 blastocysts with and without Ilo treatment (D2; R = 3; Con; n = 56, Ilo; n = 43, D6; R = 3; Con; n = 42, Ilo; n = 51). (**C**) Representative fluorescence images of day 2 embryos and day 6 blastocysts stained with CMF2HC for GSH detection in the indicated groups. Scale bar = 100 μm. (**D**) Quantification of GSH fluorescence intensity day 2 embryos and day 6 blastocysts with and without Ilo treatment (D2; R = 3; Con; n = 75, Ilo; n = 70, D6; R = 3; Con; n = 75, Ilo; n = 70). Significant differences are represented by different letters (*p* < 0.05).

To evaluate how Ilo affects the redox status of porcine embryos, intracellular ROS and GSH were quantified at both the 4-cell and blastocyst stages. Intracellular ROS levels were significantly decreased in Ilo-treated embryos at both stages compared to control groups (Figure 2A,B). In contrast, Ilo-treated embryos showed markedly elevated GSH levels at both developmental stages (Figure 2C,D).

### 3.3. Ilo Activates the Nrf2/Keap1 Pathway in Porcine PA Embryos

**Figure 3:**
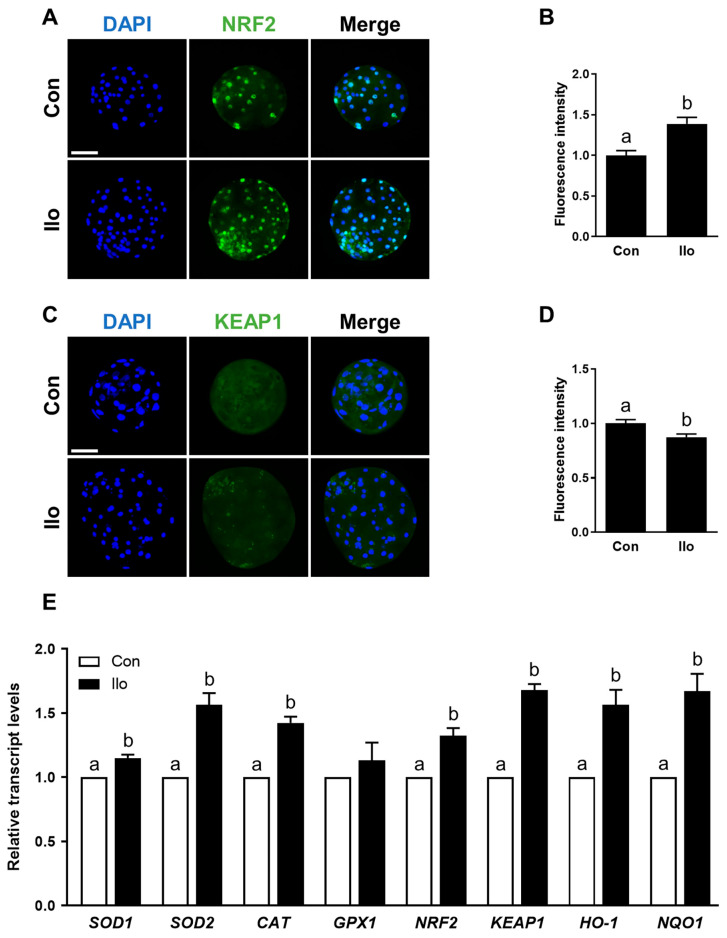
Effects of Iloprost (Ilo) treatment on activation of the Nrf2/Keap1 signaling pathway in porcine blastocysts. (**A**) Representative immunofluorescence images of Nrf2 expression in blastocysts from control and Ilo-treated group. DAPI (blue), Nrf2 (green). Scale bar = 100 μm. (**B**) Quantification of Nrf2 fluorescence intensity in the indicated groups (R = 3; Con; n = 34, Ilo; n = 33). (**C**) Representative immunofluorescence images of Keap1 expression in blastocysts. DAPI (blue), Keap1 (green). Scale bar = 100 μm. (**D**) Quantification of Keap1 fluorescence intensity in the indicated groups (R = 3; Con; n = 38, Ilo; n = 32). (**E**) Relative expression levels of Nrf2/Keap1 signaling pathway-related genes in day 6 blastocysts with and without Ilo treatment (R = 3). Significant differences are represented by different letters (*p* < 0.05).

Given that the Nrf2/Keap1 axis serves as a central regulator of cellular antioxidant responses, we examined whether Ilo modulates this pathway in porcine embryos. Immunofluorescence analysis revealed a marked increase in Nrf2 abundance in the Ilo-treated embryos, whereas Keap1 signals in the cytoplasm showed a noticeable decline (Figure 3A–D). To evaluate the functional consequence of Nrf2/Keap1 activation, we further analyzed the mRNA transcript levels of antioxidant-related genes that are known to be regulated by this pathway. qRT-PCR results showed that several major antioxidant-related transcripts—such as *SOD1*, *SOD2*, *CAT*, *HO-1*, *NQO1*, as well as *NRF2* and *KEAP1*—were markedly elevated in the Ilo-treated embryos (Figure 3E). Thus, Ilo treatment clearly activates the Nrf2/Keap1 pathway and its antioxidant gene network in porcine embryos.

### 3.4. Brusatol Impairs the Developmental Competence of Porcine PA Embryos

**Figure 4:**
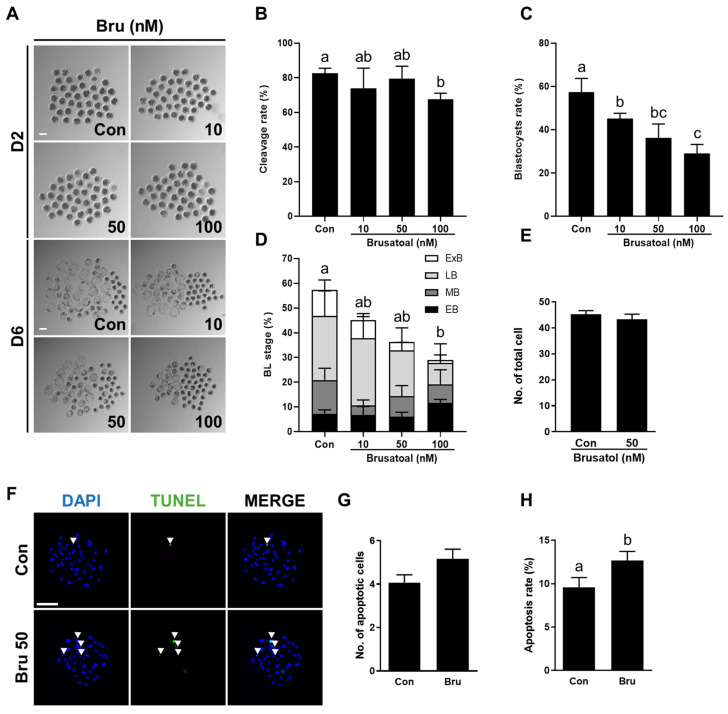
Effects of brusatol treatment on early development of parthenogenetically activated porcine embryos. (**A**) Representative bright-field images of embryos at day 2 (upper, cleaved embryos) and day 6 (lower, blastocysts) after PA with varying concentrations of brusatol. Scale bar = 100 μm. (**B**) Cleavage rate and (**C**) blastocyst formation rates of embryos in the indicated groups (R = 3; n = 69). (**D**) Proportions of different stages of blastocysts (R = 3; n = 69). (**E**) Quantification of total cell numbers in the control and brusatol-treated group (R = 3; Con; n = 53, brusatol; n = 53). (**F**) Representative images of TUNEL-stained blastocysts on day 6. Embryos were subjected to TUNEL (green, white arrow) and nuclear staining (blue). Bar = 100 μm. (**G**,**H**) Quantification of the number and proportion of apoptotic cells in the indicated groups (R = 3; Con; n = 53, brusatol; n = 53). Significant differences are represented by different letters (*p* < 0.05).

To examine the effect of brusatol, a well-known Nrf2 inhibitor, on porcine embryo development, PA embryos were cultured in IVC medium containing different concentrations of brusatol (0, 10, 50, and 100 nM) for 6 days. Brusatol treatment did not affect the cleavage rate (Figure 4A,B), but significantly decreased blastocyst formation in a concentration-dependent manner (Figure 4A,C, Table S5). The proportion of expanded blastocysts was also significantly decreased with increasing brusatol concentration (Figure 4D, Table S6). Moreover, blastocysts exposed to brusatol exhibited fewer cells overall relative to the untreated group, although this reduction was not statistically significant (Figure 4E, Table S7). The apoptosis rate was significantly higher in the brusatol-treated group compared with the control (Figure 4F–H, Table S7). Based on these dose-dependent effects, 50 nM brusatol was selected for subsequent experiments.

### 3.5. Ilo Attenuates Impairments Induced by Brusatol

**Figure 5:**
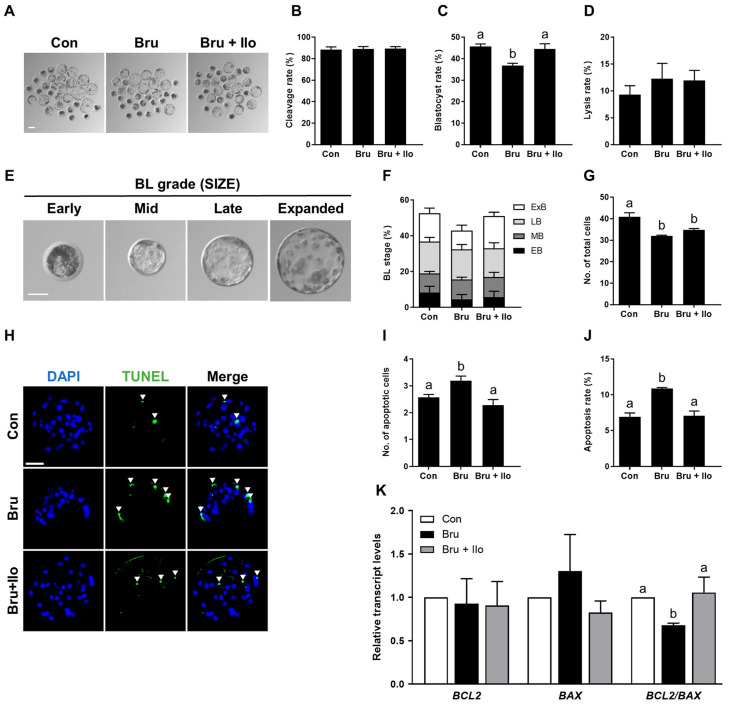
Iloprost (Ilo) treatment restores embryo development impaired by brusatol. (**A**) Representative bright-field images of blastocysts cultured in the indicated groups. scale bar = 100 μm. (**B**,**C**) Cleavage rate and blastocyst formation rate in embryos treated with brusatol alone or in combination with Ilo (R = 7; n = 227). (**D**) Proportion of lysed embryos in each group (n = 227). (**E**) Classification of blastocysts based on morphological stage. Scale bar = 50 μm. (**F**) Distribution of blastocysts according to developmental stage (R = 7; n = 227). (**G**) Quantification of total cell numbers in embryos treated with brusatol alone or in combination with Ilo (R = 7; n = 227). (**H**) Representative images of TUNEL-stained blastocysts on day 6. Embryos were subjected to TUNEL (green, white arrow) and nuclear staining (blue). Scale bar = 100 μm. (**I**,**J**) Quantification of the number and proportion of apoptotic cells in the indicated groups (R = 3; Con; n = 50, brusatol; n = 45, brusatol + Ilo; n = 53). (**K**) Relative expression levels of apoptosis-related genes (*BAX* and *BCL2*) and the *BCL2/BAX* ratio in blastocysts (R = 6). Significant differences are represented by different letters (*p* < 0.05).

To investigate whether Ilo can mitigate the detrimental effects of brusatol-induced inhibition of the Nrf2/Keap1 pathway on porcine embryo development, PA embryos were cultured in IVC medium containing brusatol with or without Ilo (1 μM) for 6 days. While cleavage rates did not differ significantly among groups, co-treatment with Ilo restored the blastocyst formation rate suppressed by brusatol (Figure 5A–D, Table S8). The proportion of lysed embryos did not differ significantly among the groups (Figure 5D). Co-treatment with Ilo tended to restore the proportion of expanded blastocysts reduced by brusatol, but the difference was not statistically significant (Figure 5E,F, Table S9). Total cell numbers were also lower in the brusatol-treated group and showed a tendency to increase with Ilo co-treatment, although the difference was not statistically significant (Figure 5G, Table S8). TUNEL assays showed that brusatol treatment significantly increased the apoptosis rate compared with the control, and this effect was markedly attenuated by Ilo treatment (Figure 5H–J, Table S10). Expression levels of apoptosis-related genes were further examined by qRT-PCR. The pro-apoptotic gene *BAX* showed a numerical increase in the brusatol-treated embryos, but this difference was not statistically significant (Figure 5K). In contrast, *BCL2* expression did not differ significantly among the groups (Figure 5K). Consequently, the *BCL2*/*BAX* ratio was significantly decreased by brusatol and subsequently restored by Ilo (Figure 5K).

### 3.6. Ilo Alleviates Oxidative Stress Induced by Brusatol

**Figure 6:**
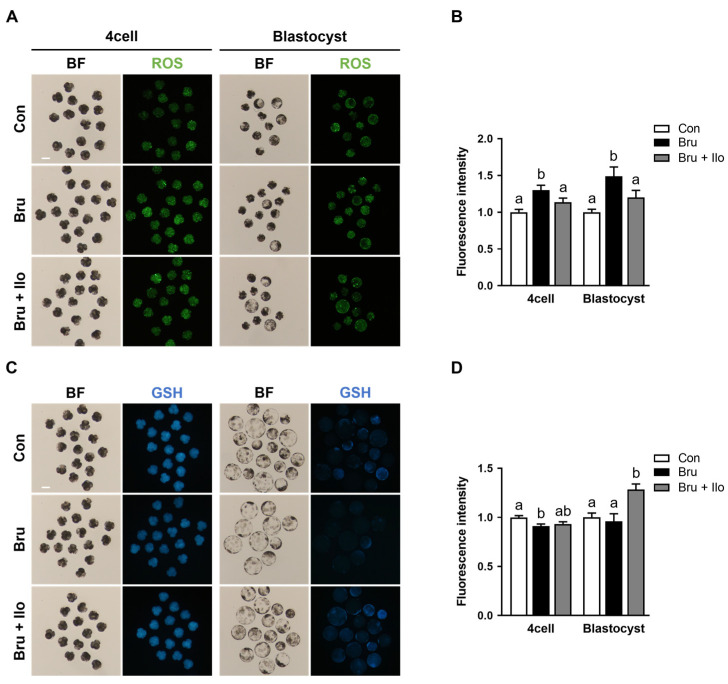
Iloprost (Ilo) treatment alleviates oxidative stress induced by brusatol. (**A**) Representative fluorescence images of day 2 embryos and day 6 blastocysts stained with CM-H2DCFDA (green) for ROS detection in embryos treated with brusatol alone or in combination with Ilo. Scale bar = 100 μm. (**B**) Quantification of ROS fluorescence intensity in day 2 embryos and day 6 blastocysts in the indicated groups. (D2; R = 3; Con; n = 46, brusatol; n = 48, brusatol + Ilo; n = 45, D6; R = 3; Con; n = 30, brusatol; n = 27, brusatol + Ilo; n = 26). (**C**) Representative fluorescence images of day 2 embryos and day 6 blastocysts stained with CMF2HC (blue) for GSH detection in embryos treated with Brusatol alone or in combination with Ilo. Scale bar = 100 μm. (**D**) Quantification of GSH fluorescence intensity in day 2 embryos and day 6 blastocysts in the indicated groups. (D2; R = 3; Con; n = 34, brusatol; n = 44, brusatol + Ilo; n = 34, D6; R = 3; Con; n = 50, brusatol; n = 22, brusatol + Ilo; n = 29). Significant differences are represented by different letters (*p* < 0.05).

To investigate whether Ilo treatment could mitigate oxidative stress resulting from Nrf2 inhibition, intracellular levels of ROS and GSH were measured in porcine PA embryos at both the 4-cell stage and the blastocyst stage. Brusatol treatment significantly increased ROS levels at both developmental stages compared with the control group, whereas co-treatment with Ilo restored ROS levels to those observed in the control group (Figure 6A,B). Conversely, intracellular GSH levels were decreased in brusatol-treated embryos (Figure 6C,D). Ilo supplementation effectively restored this reduction, resulting in significantly higher GSH levels in both 4-cell stage embryos and blastocysts compared with the brusatol-treated embryos (Figure 6C,D). These findings suggest that Ilo helps restore redox balance by reducing oxidative stress and restoring antioxidant capacity in early porcine embryos.

### 3.7. Ilo Restores Nrf2 Signaling Activity in Brusatol-Treated Blastocysts

**Figure 7:**
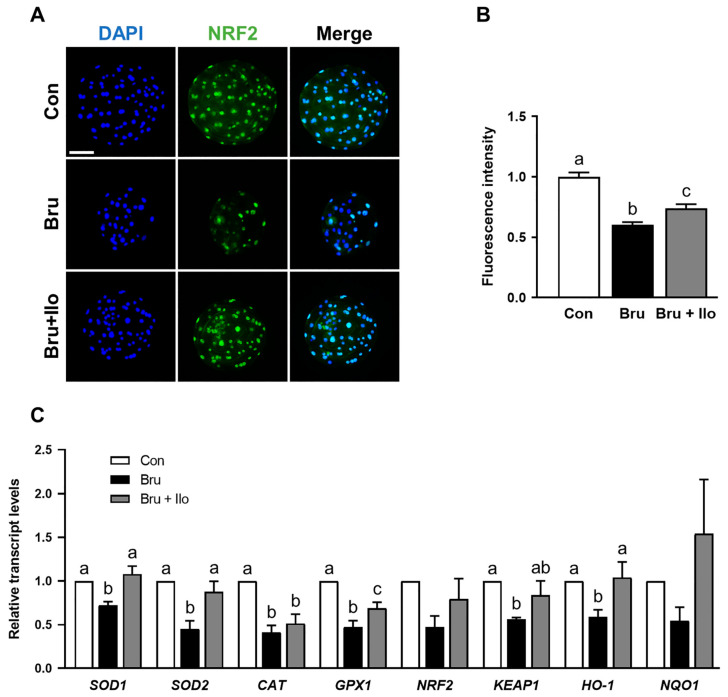
Iloprost (Ilo) restores Nrf2 nuclear localization and antioxidant gene expression suppressed by brusatol in porcine blastocysts. (**A**) Representative immunofluorescence images of Nrf2 expression in blastocysts treated with brusatol alone or in combination with Ilo. DAPI (blue), Nrf2 (green). Scale bar = 100 μm. (**B**) Quantification of Nrf2 fluorescence intensity in the indicated groups (R = 3; Con; n = 56, brusatol; n = 56, brusatol + Ilo; n = 43). (**C**) Relative expression levels of Nrf2/Keap1 signaling pathway-related genes in day 6 blastocysts in the indicated groups (R = 3). Significant differences are represented by different letters (*p* < 0.05).

Finally, we examined Nrf2 protein localization under Nrf2 inhibition to evaluate the effect of Ilo treatment on Nrf2 signaling pathway, with a specific focus on its interaction with the inhibitor brusatol treatment. Given that nuclear translocation is critical for Nrf2 activation [29], we focused on its abundance within the nucleus. Brusatol treatment, as expected, markedly reduced nuclear Nrf2 abundance compared with the control group, whereas co-treatment with Ilo significantly rescued nuclear localization (Figure 7A,B). Consistent with this, qRT-PCR analysis showed that expression of Nrf2/Keap1 pathway-related genes was upregulated in the Ilo co-treatment group compared with brusatol treatment alone (Figure 7C). Collectively, these findings suggest that Ilo facilitates Nrf2 activation, as evidenced by restored localization and increased antioxidant gene expression.

## 4. Discussion

Preimplantation embryos are regulated by various bioactive factors secreted within the oviductal environment [30]. PGI_2_ is synthesized in the oviduct via cyclooxygenase and PGI_2_ synthase, particularly in oviductal epithelial and smooth muscle cells [10]. In mammalian embryos, the presence of IP receptors on the membrane and nuclear peroxisome proliferator-activated receptor δ suggests that PGI_2_ can directly bind to embryonic cells, thereby supporting early embryonic development in the oviductal environment [31,32]. Previous studies have shown that the stable PGI_2_ analogue, Ilo, enhances early embryonic development in multiple mammalian species [28]. In mice, Ilo treatment during preimplantation improved implantation, and it increased blastocyst formation in cattle and pigs [27,33]. Ilo has also been shown to exert antioxidant effects in different tissues. For example, in pulmonary microvascular endothelial cells, Ilo inhibited ROS production, while in ischemia-reperfusion injury models of skeletal muscle and liver, it reduced lipid peroxidation (MDA) and injury-related enzymes (CK, LDH), thereby protecting tissue integrity through antioxidant defense mechanisms [34,35]. Although PGI_2_ has been associated with early embryo development, its antioxidant effects in porcine embryos have not been clearly defined. In this study, we primarily utilized PA porcine embryos as our experimental model. This approach allowed us to minimize variability potentially introduced by sperm factors in fertilized embryos, thereby enabling a more direct and focused assessment of Ilo’s impact on oocyte-derived embryonic competence and its underlying cellular mechanisms. We demonstrate that Ilo decreases ROS accumulation, increases GSH levels, and enhances blastocyst quality, suggesting that Ilo can mimic the protective oviductal environment under in vitro conditions.

Although ROS play essential roles in intracellular signaling, their excessive accumulation causes oxidative damage to lipids, proteins, and DNA, leading to impaired cell viability and function [36,37]. Thus, maintaining redox homeostasis is crucial for normal embryonic development. In vivo, oviductal epithelial cells secrete antioxidant enzymes such as glutathione peroxidase, superoxide dismutase, and catalase, which help sustain a favorable microenvironment for fertilized eggs [38,39]. Notably, PGI_2_ concentrations have been shown to increase markedly in human oviductal epithelial cells 2 to 3 days after fertilization, suggesting that PGI_2_ plays a pivotal role in establishing a physiological oviductal environment that protects early embryos and supports their development [40]. This protective effect may be closely associated with the antioxidant capacity of PGI_2_. In fact, Ilo has been shown to exert antioxidant effects in several pathophysiological conditions such as pulmonary hypertension and cardiovascular diseases [41,42]. Our results confirmed that the antioxidant function of PGI_2_ can be recapitulated in the developmental environment of porcine embryos, thereby improving developmental competence. Thus, Ilo may serve as a supplemental bioactive factor to improve in vitro culture conditions by mimicking the antioxidant environment of the oviduct. Mechanistically, Ilo binds to the PGI_2_ receptor expressed on vascular smooth muscle cells and platelets, triggering adenylate cyclase activation via the Gs protein, and subsequently increasing intracellular cAMP levels [43]. Elevated cAMP activates protein kinase A (PKA), which phosphorylates anti-apoptotic proteins and regulates antioxidant signaling cascades. PKA activation has been reported to stabilize Nrf2 and promote its nuclear translocation, leading to transcription of antioxidant-related genes under oxidative stress [44]. Consistent with this, our study showed that Ilo treatment enhanced nuclear localization of Nrf2 and upregulated expression of Nrf2-regulated genes, while reducing Keap1 levels. These findings suggest that Ilo enhances embryonic development at least in part through activation of the Nrf2/Keap1 pathway, which improves cellular defense against oxidative stress [45,46].

Our findings support the view that Ilo promotes nuclear accumulation of Nrf2 and reduces Keap1 abundance, consistent with the Nrf2/Keap1 signaling pathway. Although we did not directly assess Keap1 ubiquitination or proteasomal degradation, the observed changes in Nrf2 localization and antioxidant gene expression suggest that Ilo enhances antioxidant defenses in porcine embryos [47]. Such activation of the Nrf2/Keap1 axis is likely to contribute to improved cell survival and developmental competence by reducing oxidative damage in the embryo [48]. In this study, activation of the Nrf2/Keap1 pathway was primarily assessed at the transcriptional level and through immunofluorescence-based nuclear accumulation, which significantly contributes to elucidating the molecular mechanism by which Ilo induces antioxidant responses in early embryos. However, this study did not directly validate the functional activity of the Nrf2/Keap1 pathway at the protein level, such as via Western blotting, which will require validation in future research. Nevertheless, the consistent findings regarding transcriptional regulation and increased nuclear localization of Nrf2 provide important evidence that Ilo activates antioxidant signaling in early porcine embryos. While Ilo has been reported to improve embryo development in mice, cattle, and porcine, its functional association with the Nrf2/Keap1 pathway in porcine embryos has not been clearly established [27,33].

The functional relevance of this pathway was further confirmed using brusatol, a selective Nrf2 inhibitor that promotes Nrf2 ubiquitination and degradation [49]. Brusatol treatment significantly reduced blastocyst formation, cell number, and cell viability, while increasing apoptosis, highlighting the critical role of Nrf2 in early embryonic development. Notably, co-treatment with Ilo attenuated these detrimental effects, restoring ROS and GSH levels, blastocyst formation, and Nrf2 nuclear localization. This provides additional support that the beneficial effects of PGI_2_ analogues are mediated through the Nrf2/Keap1 pathway.

Collectively, our results indicate that Ilo functions as an antioxidant regulator in porcine embryos by activating the Nrf2/Keap1 pathway. Based on these findings, we propose a conceptual model in which Ilo alleviates oxidative stress and enhances embryo viability through Nrf2/Keap1 activation. However, an important limitation of this study is that it used only PA embryos. PA embryos inherently lack paternal genetic contributions and exhibit well-documented abnormalities in genomic imprinting, epigenetic regulation, and developmental dynamics compared with fertilized (IVF) embryos [50]. These characteristics may alter metabolic activity, redox reactions, and apoptosis-related pathways, implying that the biological behavior of PA embryos may not fully represent that of fertilized embryos. Therefore, generalizing our findings to IVF-derived or in vivo embryos is limited and should be interpreted with caution. Despite these intrinsic limitations, PA embryos provide a consistent and controlled model for investigating oocyte-derived regulatory mechanisms without confounding paternal factors. This model is particularly useful for analyzing cellular responses to environmental stressors such as oxidative stress and antioxidant treatments [51]. The general consistency observed across multiple developmental parameters and molecular markers in this study, including ROS and GSH levels, Nrf2 nuclear localization, antioxidant gene expression, and apoptosis, supports the rationale for using PA embryos in mechanistic studies of oxidative stress responses. This is particularly relevant given that porcine embryos are more susceptible to oxidative stress than those of other species and often exhibit reduced developmental quality under in vitro conditions [52,53]. By alleviating ROS accumulation and enhancing blastocyst quality, Ilo may act as a supplemental factor that mimics the physiological protection of the oviduct. These findings not only provide mechanistic insight into how PGI_2_ signaling supports preimplantation development but also suggest potential applications of PGI_2_ analogues for optimizing embryo culture systems in assisted reproductive technologies.

## 5. Conclusions

This study demonstrates that Ilo functions as an antioxidant regulator in porcine embryos by activating the Nrf2/Keap1 signaling pathway. Ilo effectively alleviated ROS accumulation induced by in vitro culture conditions and enhanced both blastocyst formation and cell viability, highlighting its protective role against oxidative stress. These findings suggest that PGI_2_, secreted by oviductal epithelial cells, may act as a physiological modulator that safeguards embryos during early development.

A deeper understanding of such endogenous protective systems can provide valuable insights for improving current in vitro embryo culture systems and optimizing assisted reproductive technologies. Future studies should clarify how IP receptor-mediated signaling functionally interacts with the Nrf2/Keap1 pathway. Such investigations may serve as a foundational basis for regenerative approaches or the development of advanced culture systems aimed at precisely mimicking the physiological in vivo environment under in vitro conditions.

## Data Availability

The original contributions presented in this study are included in the article/Supplementary Material. Further inquiries can be directed to the corresponding author.

## Supplementary Materials

The following supporting information can be downloaded at: https://www.mdpi.com/article/10.3390/antiox14121493/s1, Table S1: Primer sequences used for qRT-PCR; Table S2: Effect of Ilo on porcine embryonic developmental competence; Table S3: Effect of Ilo on blastocyst of expansion; Table S4: Effects of Ilo on cell survival in blastocysts; Table S5: Effect of brusatol on embryonic developmental competence; Table S6: Effect of brusatol on blastocyst of expansion; Table S7: Effects of brusatol on cell survival in blastocysts; Table S8: Recovery effect of Ilo treatment on embryonic development in brusatol-treated embryos; Table S9: Recovery effect of Ilo treatment on blastocyst of expansion in brusatol-treated embryos; Table S10: Recovery effect of Ilo treatment on cell survival in blastocyst in brusatol-treated embryos.

## Abbreviations

The following abbreviations are used in this manuscript:

PGI_2_	Prostacyclin
cAMP	Cyclic amp
Ilo	Iloprost
ROS	Reactive oxygen species
IVC	In vitro culture
Nrf2	Erythroid 2 related factor 2
Keap1	Kelch like ECH associated protein 1
ARE	Antioxidant response element
GPX	Glutathione peroxidase
SOD	Superoxide dismutase
CAT	Catalase
HO-1	Heme oxygenase-1
IVM	In vitro maturation
COCs	Cumulus-oocyte complexes
BSA	Bovine serum albumin
PA	Parthenogenetic activation
DPBS	Dulbecco’s phosphate-buffered saline
PZM	Porcine zygote medium
DMSO	Dimethyl sulfoxide
TUNEL	Terminal deoxynucleotidyl transferase-mediated dUTP-digoxygenin nick end-labeling
PVA-PBS	0.1% Polyvinyl alcohol
RT	Room temperature
DAPI	4′,6-Diamidino-2-phenylindole
GSH	Glutathione
qRT-PCR	Quantitative real-time polymerase chain reaction
PKA	Protein kinase A
