# Supplementary material for: Iloprost, a Prostacyclin Analogue, Alleviates Oxidative Stress and Improves Development of Parthenogenetic Porcine Embryos via Nrf2/Keap1 Signaling
Source: Antioxidants (Basel). 2025 Dec 12;14(12):1493. doi: 10.3390/antiox14121493 (PMC12729714; doi:10.3390/antiox14121493)
Supplement: Supplementary file 1 [file antioxidants-14-01493-s001.zip › Supplemental data_Choi et al..pdf]

Table S1 Primer sequences used for qRT-PCR

Gene	Primer sequences	GenBank accession no.	Product size (bp)
OCT4	F: 5’- AGTGAGAGGCAACCTGGAGA -3’<br>R: 5’- ACTGCTTGATCGTTTGCCCT -3’	NM_001113060.1	151
CDX2	F: 5’- GGCAGCCAAGTGAAAACCAG -3’<br>R: 5’- GCCTTTCTCCGAATGGTGAT -3’	NM_001278769.1	119
BAX	F: 5’- CGATCTCGAAGGAAGTCCAG -3’<br>R: 5’- AAGCGCATTGGAGATGAACT -3’	XM_003127290.5	251
BCL2	F: 5’- AGGGCATTCAGTGACCTGAC -3’<br>R: 5’- CGATCCGACTCACCAATACC -3’	NM_214285.1	196
SOD1	F: 5’- GGTGGGCCAAAGGATCAAGA -3’<br>R: 5’- TACACAGTGGCCACACCATC -3’	NM_001190422.1	80
SOD2	F: 5’- GGTGGAGGCCACATCAATCA -3’<br>R: 5’- AACAAGCGGCAATCTGCAAG -3’	NM_214127.2	220
CAT	F: 5’- TGTACCCGCTATTCTGGGGA -3’<br>R: 5’- TCACACAGGCGTTTCCTCTC -3’	NM_214301.2	119
GPX1	F: 5’- TGGACATCAGGAAAATGCCAAG -3’<br>R: 5’- GTGAGCATTTGCGCCATTCA -3’	NM_214201.1	127
NRF2	F: 5’- CCTTCTGGGGATACAGTCCA -3’<br>R: 5’- CCGGGACTTATAGGCACTTC -3’	XM_005671981.3	110
KEAP1	F: 5’- GCCTCATCGAGTTCGCTTAC -3’<br>R: 5’- CACGGACCACACTGTCAATC -3’	NM_001114671.1	105
HO-1	F: 5’- CCTTTTGACGTGCCTTGACT -3’<br>R: 5’- GAACGAAGAGTGGCTCCAAC -3’	NM_001004027.1	114
NQO1	F: 5’- GTCTTTCTGTGGGCCATCAC-3’<br>R: 5’- CGAAAGCAAGTCAAGGAAGG -3’	NM_001159613.1	146
H2A	F: 5’- AGTTTCCTGTGGGTCGAGTG -3’<br>R: 5’- TGCGAGTCTTCTTGTTGTC -3’	XM_021083382.1	162

F: forward, R: reverse

Table S2 Effect of Ilo on porcine embryonic developmental competence

Groups	No. of embryos examined	Cleavage, %	Blastocyst, %	Total cell number

Con	112	104 (92.5 ± 1.2)	48 (43.4 ± 1.8)[a]	36 ± 2.8[a]
Ilo	119	111 (93.4 ± 0.6)	62 (52.4 ± 1.2)[b]	45.2 ± 3.0[b]

Data are the mean ± SEM, and values with different superscript letter within a column differ significantly ($P < 0.05$). Ilo; Iloprost

Table S3 Effect of Ilo on blastocyst of expansion

Groups	No. of blastocysts examined	Proportion of blastocysts developed to the following stages (%)			
		Early	Middle	Large	Expanded
Con	64	23.9 ± 1.7 [a]	38.9 ± 7.4	16.3 ± 6.0	20.9 ± 0.8 [a]
Ilo	81	16.2 ± 2.1 [b]	20.7 ± 6.2	25.1 ± 3.8	38.1 ± 3.8 [b]

Data are the mean ± SEM, and values with different superscript letter within a column differ significantly ($p < 0.05$).

Table S4 Effects of Ilo on cell survival in blastocysts

**Groups**	**No. of blastocysts examined**	**No. of TUNEL-positive cells**	**Apoptosis, %**
Con	47	3.6 ± 0.3 [a]	13.3 ± 1.4[a]
Ilo	46	2.5 ± 0.3 [b]	9.2 ± 1.1[b]

Data are the mean ± SEM, and values with different superscript letter within a column differ significantly ($P < 0.05$)

Table S5 Effect of brusatol on embryonic developmental competence

**Bru, nM**	**No. of embryos examined**	**Cleavage, %**	**Blastocyst, %**
0	69	57 (82.6 ± 1.6) [a]	39 (57.3 ± 3.6)[a]
10	69	51 (73.9 ± 6.6) [ab]	31 (45.1 ± 1.3) [b]
50	69	55 (79.5 ± 4.1) [ab]	25 (36.1 ± 3.7) [bc]
100	69	47 (67.6 ± 1.9) [b]	20 (28.9 ± 2.4)[c]

Data are the mean ± SEM, and values with different superscript letter within a column differ significantly ($P < 0.05$)

Table S6 Effect of brusatol on blastocyst of expansion

Bru, nM	No. of embryos examined	Proportion of blastocysts developed to the following stages (%)			
		Early	Middle	Large	Expanded
0	69	7.3 ± 1.5	13.5 ± 4.7	25.8 ± 5.8 [a]	10.5 ± 2.3[a]
10	69	6.7 ± 3.3	3.9 ± 2.0	27.1 ± 4.9 [a]	7.3 ± 1.5[ab]
50	69	6.03 ± 1.7	8.3 ± 4.2	18.4 ± 1.8 [ab]	3.3 ± 3.3[ab]
100	69	11.67 ± 1.3	7.5 ± 5.8	8.5 ± 4.4 [b]	1.1 ± 1.1[b]

Data are the mean ± SEM, and values with different superscript letter within a column differ significantly ($p < 0.05$).

Table S7 Effects of brusatol on cell survival in blastocysts

Groups	No. of blastocysts examined	No. of TUNEL-positive cells	Apoptosis, %	Total cell number
Con	53	4.0 ± 0.3	10.2 ± 1.1[a]	45.2 ± 1.3
Bru	53	5.1 ± 0.4	12.6 ± 1.0[b]	43.2 ± 1.9

Data are the mean ± SEM, and values with different superscript letter within a column differ significantly ($P < 0.05$)

Table S8 Recovery effect of Ilo treatment on embryonic development in brusatol treated embryos

Groups	No. of embryos examined	Cleavage, %	Blastocyst, %	Total cell number
Con	227	202 (88.3 ± 2.6)	112 (45.5 ± 1.2)[a]	41.2 ± 1.6[a]
Bru	227	200 (88.9 ± 2.3)	91 (36.8 ± 1.0)[b]	31.9 ± 0.4[b]
Bru + Ilo	227	204 (89.3 ± 1.8)	110 (44.5 ± 2.3)[a]	34.7 ± 0.7 [b]

Data are the mean ± SEM, and values with different superscript letter within a column differ significantly ($P < 0.05$)

Table S9 Recovery effect of Ilo treatment on blastocyst of expansion in brusatol treated embryos

Groups	No. of embryos examined	Proportion of blastocysts developed to the following stages (%)			
		Early	Middle	Large	Expanded
Con	227	8.1 ± 3.5	10.8 ± 1.1	17.8 ± 2.3	15.9 ± 2.8
Bru	227	4.5 ± 2.6	11.1 ± 1.2	16.7 ± 2.7	10.6 ± 3.0
Bru + Ilo	227	5.7 ± 3.3	11.2 ± 2.5	16.1 ± 3.1	18.1 ± 2.1

Data are the mean ± SEM, and values with different superscript letter within a column differ significantly ($p <$

0.05).

Table S10 Recovery effect of Ilo treatment on cell survival in blastocyst in brusatol treated embryos

Groups	No. of blastocysts examined	No. of TUNEL-positive cells	Apoptosis, %
Con	50	3.1 ± 0.0^{a}	8.2 ± 0.3^{a}
Bru	45	3.1 ± 0.1^{a}	10.8 ± 0.1^{b}
Bru + Ilo	53	2.2 ± 0.2^{b}	7.0 ± 0.6^{a}

Data are the mean ± SEM, and values with different superscript letter within a column differ significantly ($P <$ 0.05)
